# Prognostic ability of the sequential organ failure assessment score in accidental hypothermia: a multi-institutional retrospective cohort study

**DOI:** 10.1186/s13049-019-0681-8

**Published:** 2019-11-12

**Authors:** Kenji Kandori, Yohei Okada, Tasuku Matsuyama, Sachiko Morita, Naoki Ehara, Nobuhiro Miyamae, Takaaki Jo, Yasuyuki Sumida, Nobunaga Okada, Makoto Watanabe, Masahiro Nozawa, Ayumu Tsuruoka, Yoshihiro Fujimoto, Yoshiki Okumura, Tetsuhisa Kitamura, Ryoji Iiduka

**Affiliations:** 1 Department of Emergency and Critical Care Medicine, Japanese Red Cross Society, Kyoto Daini Hospital, Kyoto, Japan; 2 Department of Primary Care and Emergency Medicine, Graduate School of Medicine, Kyoto University, Kyoto, Japan; 3 Department of Emergency Medicine, Kyoto Prefectural University of Medicine, Kyoto, Japan; 4 Senri Critical Care Medical Center, Saiseikai Senri Hospital, Suita, Japan; 5 Department of Emergency, Japanese Red Cross Society, Kyoto Daiichi Red Cross Hospital, Kyoto, Japan; 6 Department of Emergency Medicine, Rakuwa-kai Otowa Hospital, Kyoto, Japan; 7 Department of Emergency Medicine, Uji-Tokushukai Medical Center, Uji, Japan; 8 Department of Emergency Medicine, North Medical Center, Kyoto Prefectural University of Medicine, Kyoto, Japan; 9 Department of Emergency and Critical Care Medicine, National Hospital Organization, Kyoto Medical Center, Kyoto, Japan; 10 Department of Emergency and Critical Care Medicine, Saiseikai Shiga Hospital, Ritto, Japan; 11 Department of Emergency and Critical Care Medicine, Kyoto Min-Iren Chuo Hospital, Kyoto, Japan; 12 Department of Emergency Medicine, Yodogawa Christian Hospital, Osaka, Japan; 13 Department of Emergency Medicine, Fukuchiyama City Hospital, Fukuchiyama, Japan; 14 Division of Environmental Medicine and Population Sciences, Department of Social and Environmental Medicine, Graduate School of Medicine, Osaka University, Osaka, Japan

**Keywords:** Accidental hypothermia, Environmental emergency, Mortality, The sequential organ failure assessment, The systemic inflammatory response syndrome

## Abstract

**Background:**

Severe accidental hypothermia (AH) is life threatening. Thus, prognostic prediction in AH is essential to rapidly initiate intensive care. Several studies on prognostic factors for AH are known, but none have been established. We clarified the prognostic ability of the Sequential Organ Failure Assessment (SOFA) score in comparison with previously reported prognostic factors among patients with AH.

**Methods:**

The J-point registry database is a multi-institutional retrospective cohort study for AH in 12 Japanese emergency departments. From this registry, we enrolled patients who were treated at the intensive care unit (ICU) in various critical care medical centers. In-hospital mortality was the primary outcome. We investigated the discrimination ability of each candidate prognostic factor and the in-hospital mortality by applying the logistic regression models with areas under the receiver operating characteristic curve (AUROC) with 95% confidence interval (CI).

**Results:**

Of the 572 patients with AH registered in the J-point registry, 220 were eligible for the analyses. The in-hospital mortality was 23.2%. The AUROC of the SOFA score (0.80; 95% CI: 0.72–0.86) was the highest among all factors. The other factors were serum potassium (0.65; 95% CI: 0.55–0.73), lactate (0.67; 95% CI: 0.57–0.75), quick SOFA (qSOFA) (0.55; 95% CI: 0.46–0.65), systemic inflammatory response syndrome (SIRS) (0.60; 95% CI: 0.50–0.69), and 5A severity scale (0.77; 95% CI: 0.68–0.84).

**Discussion:**

Although serum potassium and lactate had relatively good discrimination ability as mortality predictors, the SOFA score had slightly better discrimination ability. The reason is that lactate and serum potassium were mainly reflected by the hemodynamic state; conversely, the SOFA score is a comprehensive score of organ failure, basing on six different scores from the respiratory, cardiovascular, hepatic, coagulation, renal, and neurological systems. Meanwhile, the qSOFA and SIRS scores underestimated the severity, with low discrimination abilities for mortality.

**Conclusions:**

The SOFA score demonstrated better discrimination ability as a mortality predictor among all known prognostic factors in patients with AH.

## Background

Accidental hypothermia (AH) is an unintentional decrease in core body temperature (BT) to 35 °C or less [1]. Generally, AH happens among the elderly people, and it can be fatal, thereby suggesting an important problem in a super-aging society [2–4]. Patients with AH are at risk of fatal arrhythmias and hemodynamic collapse; therefore, they must be assessed immediately assessed to determine their severity, and a rapid and aggressive intensive care intervention should be performed. Prognostic prediction in AH is essential to rapidly initiate intensive care, thereby, saving the lives of patients having severe condition. Therefore, research on the prognosis prediction of AH is required.

Several prognostic factors, such as age, sex, activities of daily living, BT, blood potassium level, and lactate level, for AH have been extensively studied [5–20]. Recently, 5A severity scale has also been proposed [21]. However, none of the factors have been established. Meanwhile, the Sequential Organ Failure Assessment (SOFA) score is widely accepted as a severity and prognostic factor in the field of emergency and intensive care, especially in patients suffering from sepsis [22, 23].

This study aimed to clarify the prognostic ability of the SOFA score in comparison with previously reported prognostic factors in patients with AH.

## Methods

### Study design and setting

This study is a multi-institutional retrospective cohort study, and the setting was the J-point registry. The J-point registry is a database for patients with AH transferred to the emergency departments, and it was conducted in the emergency department of eight critical care medical centers (CCMCs) and four non-CCMCs in Kyoto, Osaka, and Shiga Prefecture in Japan. In Japan, CCMCs are certified by the Ministry of Health, Labour and Welfare based on emergency departments that treat patients for shock, trauma, resuscitation, and critical care which serve approximately 500,000 residents in each region; in these CCMCs, advanced treatment such as extracorporeal membrane oxygenation (ECMO) is generally available. The median annual emergency department visit volume for participating institutions was 19,651 (interquartile range [IQR], 13,281–27,554). We retrospectively identified and registered eligible patients using the International Classification of Diseases, Tenth Revision (ICD-10) code T68: “Hypothermia”, who were diagnosed during the study period, from April 1, 2011, to March 31, 2016. We excluded the patients from the registry if they or their family members explicitly refused to be included in the registry. Clinical data were extracted by emergency physicians using a predefined data extraction sheet. The collected data were rechecked by the J-point Registry Working Group members and were either confirmed or checked with the appropriate institution if concerns regarding the data’s validity are known. On the basis of these factors, 572 patients were registered in the J-point registry. The clinical research ethics committee of each institution approved the conduct of this research.

### Study patients

The subjects of this study were patients with AH enrolled in the J-point registry and treated at the intensive care unit (ICU) in eight CCMCs. The study was limited to patients admitted to ICU in order to exclude patients who died as outpatients or who were admitted without indication for an aggressive treatment at the time of first treatment.

### Data collection

Based on the medical record review of the researcher at each facility, the following baseline patient characteristics were collected: sex, age, activities of daily living before hypothermia, and a comprehensive past medical history, including cardiovascular diseases (ischemic heart diseases, heart failure, arrhythmia, hypertension, and others), neurological diseases (stroke, epilepsy, Parkinson disease or syndrome, and others), endocrine diseases (diabetes, thyroid diseases, adrenal insufficiency, and others), psychiatric diseases (chronic alcoholism, depression, schizophrenia, and others), malignant diseases, dementia, and others. With regard to in-hospital measurements, the data included were as follows: vital signs during hospital arrival [core BT, systolic blood pressure, heart rate, Glasgow (GCS), and Japan Coma Scales (JCS)], blood test [complete blood count, total bilirubin (mg/dL), and creatinine (mg/dL), serum lactate (mmol/L), potassium (mEq/L), glucose (mg/dL)], cold exposure, treatment process (external and minimally invasive rewarming methods: warming intravenous fluid, warm air, warm blanket, and others; active internal rewarming methods: lavage, intravascular rewarming device, and veno-venous and veno-arterial ECMO), and the outcome. The JCS, a grading system that evaluates disturbed consciousness, was first published in 1974 and since then has been certified as a standard field tool assessing the level of consciousness by the Japan Fire and Disaster Management Agency.

### Outcome measurements

The primary outcome of this study was in-hospital mortality.

### Statistical analysis

We described the data on patient characteristics as medians with IQRs for continuous variables and as numbers with percentages for categorical variables. For the primary analysis, we set the SOFA score and the primary outcome (in-hospital mortality) as explanatory and objective variables, respectively, and we employed the logistic regression model. The discrimination ability of the score was assessed using the area under the receiver operating characteristic curve (AUROC) curve with 95% confidence interval (CI). We also calculated the AUROC of the following prognostic factors: age, BT, serum potassium, lactate, quick SOFA (qSOFA), systemic inflammatory response syndrome (SIRS) scores and 5A severity scale. Then, we compared the individual discrimination ability of these factors. Such comparison was demonstrated because age, BT, serum potassium, and lactate are associated with the mortality of patients with AH [5, 7, 8, 11, 13–18]; in addition, the qSOFA, SIRS, and especially the SOFA score are widely accepted to assess the severity of sepsis [23, 24]. In recent years, a 5A severity scale has been developed to predict in-hospital mortality after AH. We compared the AUROC of SOFA with those of the other factors. All *P* value analyses were two-sided, and a P value of less than 0.05 was considered significant. Moreover, the statistical analysis was performed using the JMP Pro 14 for Mac (SAS Institute, Tokyo, Japan).

## Results

### Patient characteristics

**Fig. 1:**
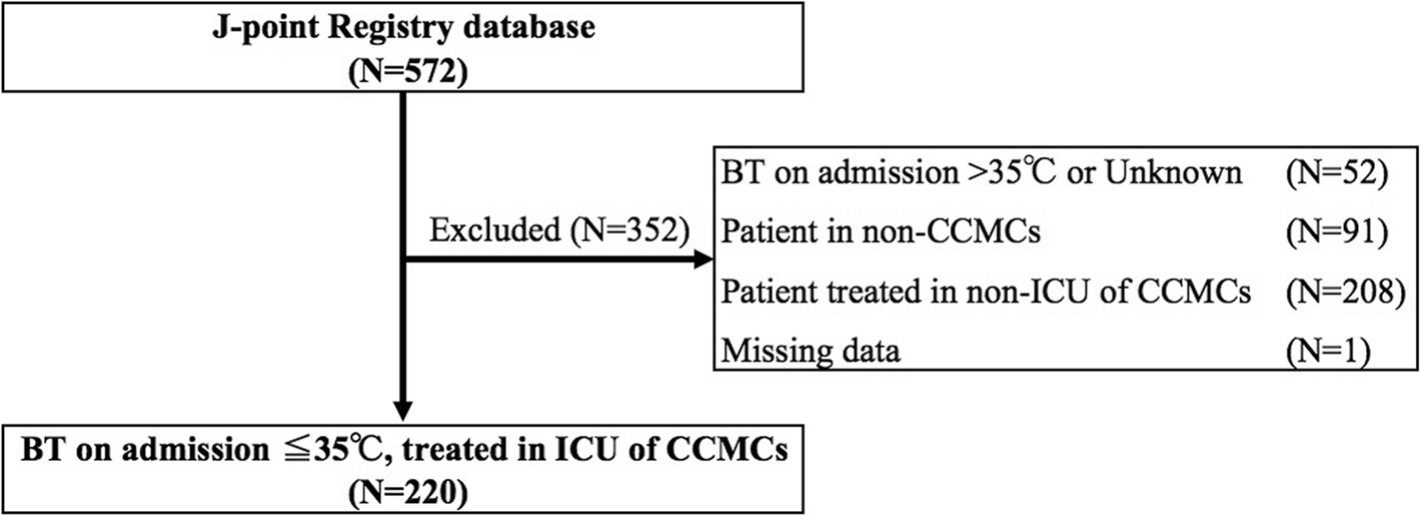
Study flowchart. BT, body temperature; CCMC, critical care medical center; ICU, intensive care unit

Of all the 572 patients with AH registered in the J-point registry database, we excluded 52 patients whose BT was above 35 °C or unknown at admission, 91 who were treated at non-CCMCs, 208 who were treated outside the ICU of CCMCs, and 1 with missing data. No one was refused to participate in the registry. Ultimately, we included 220 patients, with an overall in-hospital mortality of 23.2% (Fig. 1). The patient characteristics are shown in Table 1.

**Table 1:** Patient characteristics

Variable [number or % (IQR)]	Total patients				Survival discharge				In-hospital mortality			
	(N	=	220	)	(N	=	169	)	(N	=	51	)
Age (y.o)	77	[	64–86	]	76	[	63–86	]	80	[	70–87	]
Male sex	115	(	52.3	%)	84	(	49.7	%)	31	(	60.8	%)
Comorbidities												
Cardiovascular diseases	103	(	46.8	%)	76	(	45.0	%)	27	(	52.9	%)
Neurological diseases	31	(	14.1	%)	26	(	15.4	%)	5	(	9.8	%)
Endocrine diseases	51	(	23.2	%)	40	(	23.7	%)	11	(	21.6	%)
Psychiatric diseases	56	(	25.5	%)	47	(	27.8	%)	9	(	17.6	%)
Malignant diseases	19	(	8.6	%)	15	(	8.9	%)	4	(	7.8	%)
Dementia	26	(	11.8	%)	19	(	11.2	%)	7	(	13.7	%)
Other	35	(	15.9	%)	28	(	16.6	%)	7	(	13.7	%)
Vital signs												
Body Temperature, °C	30.4	[	27.3–32.1	]	30.5	[	27.3–32.2	]	29.6	[	26.3–32.1	]
Heart rate, /min	65	[	43–86	]	68	[	47–89	]	57	[	34–80	]
Systolic blood pressure, mmHg	107	[	80–137	]	109	[	86–136	]	96	[	56–141	]
Respiratory rate, /min	18	[	13–24	]	20	[	14–24	]	15	[	10–23	]
Glasgow coma scale	10	[	6–13	]	10	[	7–13	]	6	[	3–10	]
Laboratory results												
Potassium, mmol/L	4.1	[	3.6–4.9	]	4.0	[	3.5–4.7	]	4.6	[	3.8–5.8	]
Lactate, mmol/L	3.6	[	1.6–8.1	]	3.2	[	1.5–6.4	]	7.1	[	3.1–9.5	]
External and minimally invasive rewarming												
Warm intravenous fluid	189	(	85.9	%)	142	(	84.0	%)	47	(	92.2	%)
Forced warm air	42	(	19.1	%)	31	(	18.3	%)	11	(	21.6	%)
Warm blanket	144	(	65.5	%)	112	(	66.3	%)	32	(	62.7	%)
Other	50	(	22.7	%)	35	(	20.7	%)	15	(	29.4	%)
Active internal rewarming												
Lavage	20	(	9.1	%)	15	(	8.9	%)	5	(	9.8	%)
Intravascular	3	(	1.4	%)	2	(	1.2	%)	1	(	2.0	%)
Hemodialysis	20	(	9.1	%)	17	(	10.1	%)	3	(	5.9	%)
VV-ECMO	2	(	0.9	%)	2	(	1.2	%)	0	(	0.0	%)
VA-ECMO	17	(	7.7	%)	9	(	5.3	%)	8	(	15.7	%)
Illness severity												
qSOFA	2	[	1–2	]	2	[	1–2	]	2	[	1–2	]
SOFA	5	[	3–8	]	5	[	3–7	]	8	[	7–11	]
SIRS	2	[	1–3	]	2	[	2–3	]	2	[	1–3	]
5A severity scale	4	[	3–5	]	3	[	2–4	]	5	[	4–6	]

Values are median (interquartile range) or number (percentage)

*IQR* Interquartile range, *VV-ECMO* Veno-venous Extracorporeal membrane oxygenation, *VA-ECMO* Veno-venous Extracorporeal membrane oxygenation, *qSOFA* quick SOFA, *SOFA* Sequential Organ Failure Assessment, *SIRS* Systemic inflammatory response syndrome

### Outcome

**Fig. 2:**
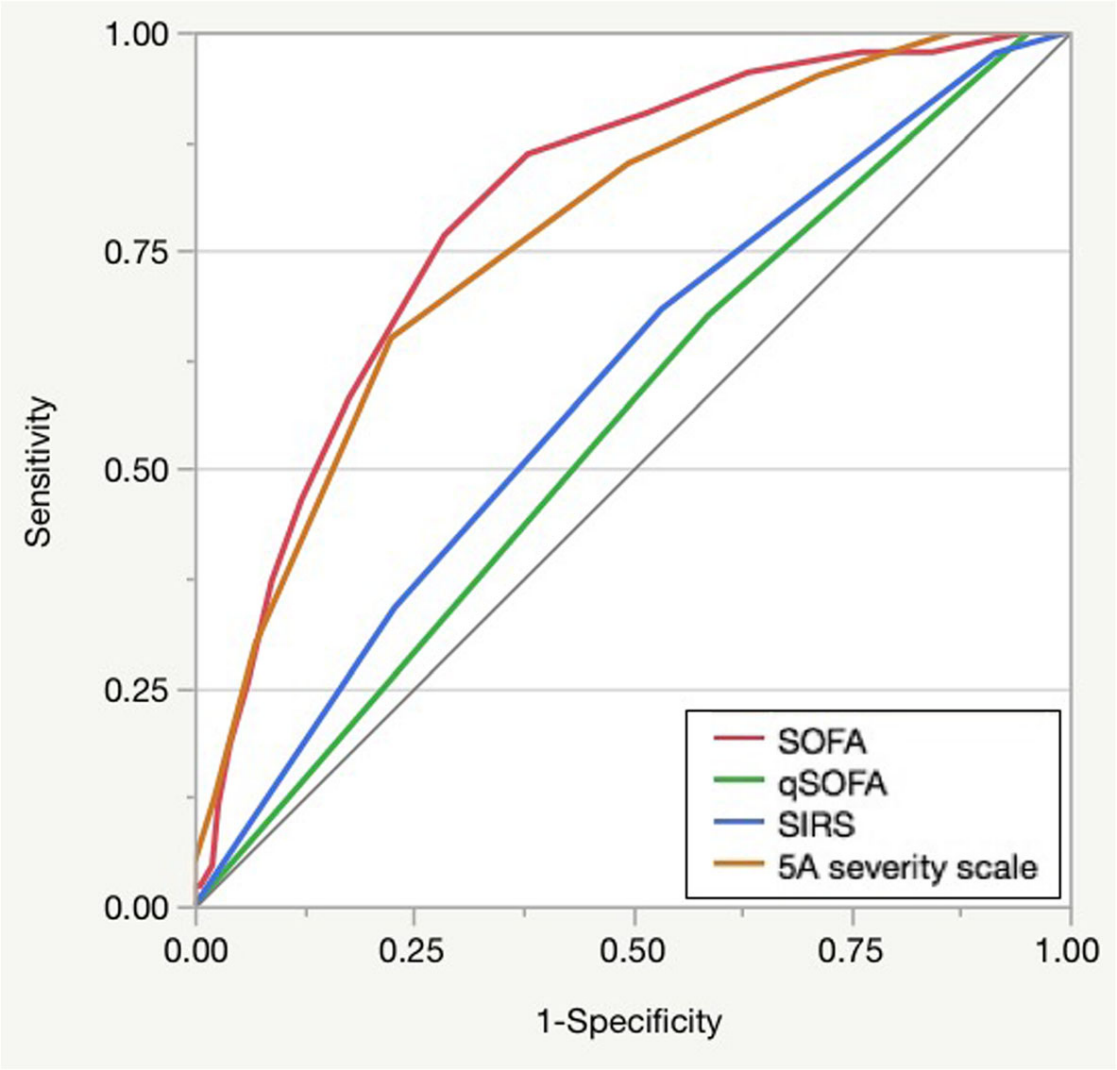
Area under the receiver operating characteristic curves for SOFA, qSOFA, SIRS, and 5A severity scale to discriminate the capacity for in-hospital mortality of accidental hypothermia

Logistic regression analysis showed that the AUROC in the SOFA, qSOFA, SIRS, and a 5A severity scale were 0.80 (95% CI: 0.72–0.86), 0.55 (95% CI: 0.46–0.65), 0.60 (95% CI: 0.50–0.69), and 0.77 (95% CI: 0.68–0.84), respectively. In addition, the AUROC of the other factors was as follows: age, 0.56 (95% CI: 0.47–0.64); BT, 0.53 (95% CI: 0.44–0.62); serum potassium, 0.65 (95% CI: 0.55–0.73); and lactate, 0.67 (95% CI: 0.57–0.75). Therefore, the AUROC of the SOFA score was the highest except 5A severity scale (Table 2 and Fig. 2). The AUROC of the SOFA score and a 5A severity scale was almost same.

**Table 2:** AUROC value for discrimination ability for in-hospital mortality of each variable

			Between-Group Difference		
Variables	AUROC	95% CI	SOFA vs variable	95% CI	*P* value
Age	0.56	[0.46–0.64]	0.24	[0.11–0.37]	<.001
Body Temperature	0.53	[0.44–0.62]	0.27	[0.15–0.39]	<.001
Laboratory results					
Potassium	0.65	[0.55–0.73]	0.15	[0.04–0.26]	<.001
Lactate	0.66	[0.57–0.75]	0.13	[0.02–0.25]	0.02
Illness severity					
SOFA	0.80	[0.72–0.86]			
qSOFA	0.55	[0.46–0.64]	0.25	[0.15–0.34]	<.001
SIRS	0.60	[0.50–0.69]	0.20	[0.09–0.32]	<.001
5A severity scale	0.77	[0.68 0.84]	0.03	[−0.07–0.13]	0.54

*AUROC* Area under the receiver operationg characteristic curve, *CI* Confidence interval, *qSOFA* quick SOFA, *SOFA* Sequential organ failure assessment, *SIRS* Systemic inflammatory response syndrome criteria

## Discussion

To the best of our knowledge, this study is the first to evaluate the performance of the SOFA score in patients with AH. In particular, we highlighted that the SOFA score had better ability to predict mortality in AH among all the reported prognostic factors.

We suggested possible explanation regarding the good performance of the SOFA score in predicting mortality among patients with AH. In this study, the SOFA score demonstrated good discrimination ability in predicting AH mortality. In previous studies, factors such as age, gender, BT at admission, potassium, and lactate were considered mortality predictors [5–20]. Although serum potassium and lactate had relatively good discrimination abilities as mortality predictors, as shown in our study, the SOFA score was slightly better than others (Table 2). Serum lactate and potassium were mainly and only reflected the hemodynamic state, whereas the SOFA score is a comprehensive assessment of multiple organ failure including six domains; respiratory, cardiovascular, hepatic, coagulation, renal, and neurological systems. In addition, organ failure caused by hypo-perfusion to vital organ or sepsis is considered to be the possible cause of mortality in patients with moderate to severe AH. Therefore, we believe that the SOFA score can assess the severity more appropriately than serum lactate or potassium.

The discrimination abilities of the qSOFA and SIRS scores were not as high as that of the SOFA score (Fig. 2), possibly because the items, such as respiratory rate, consciousness, and BT, of these scores are not sensitive for the mortality in patients with AH. With regard to the respiratory rate, patients with AH with lower BT tend to experience bradypnea [2]. However, in the qSOFA and SIRS scores, the respiratory condition is only assessed by the presence of tachypnea. Therefore, these scores cannot evaluate the respiratory condition appropriately in patients with severe AH. In contrast, the SOFA score uses PaO_2_/F_I_O_2_ for the respiratory condition; this rate is independent from the respiratory rate. Thus, the SOFA score can more accurately assess the respiratory condition in patients with AH than the qSOFA and SIRS scores. Regarding consciousness, the change in mental status is evaluated in the qSOFA; however, only binary variables (yes or no) are required as answers, and qSOFA cannot discriminate the minor and severe disturbances of consciousness in patients with AH. Conversely, the SOFA score includes five grades by using the GCS; thus, it enables to assess consciousness more exactly than qSOFA. In the SIRS score, BT is an item evaluated as more than 38 °C or lower than 36 °C, but in patients with AH, BT is basically lower than 35 °C; thus, it cannot distinguish the severity. Therefore, the qSOFA and SIRS scores underestimated the severity of AH in this study, and their discrimination abilities for mortality were low.

The SOFA score and 5A severity scale (Table 3) had almost similar discrimination ability for predicting in-hospital mortality after AH. However, SOFA score is widely accepted; thus, it may be more useful and generalized than 5A severity scale.

**Table 3:** Variables of the SOFA and 5A score

SOFA score	5A scoring model
Respiratory system (PaO_2_/F_I_O_2_)	Age
Coagulation (Platelets)	ADL
Hepatic system (Bilirubin)	Arrest
Cardiovascular system (Hypotension)	Acidemia
Central nervous system (Glasgow Coma Scale)	Albumin
Renal system (Creatinine or urine output)	

*SOFA* Sequential organ failure assessment, *ADL* Activities of daily living

This study indicated that the SOFA score may be validated to predict in-hospital mortality and assess the severity objectively among the patients with AH admitted to ICU. Therefore, it may be helpful in selecting the appropriate patients for ICU admission and shared-decision making with the patients/their families. Generally, a scoring system is useful to generalize the severity for comparing the quality of the care in different settings or for selecting patients in clinical research [25]. Thus, in patients with AH, the SOFA score may also be useful in quality management and research.

### Limitations

This study has several limitations. First, it is a retrospective cohort study. Considering that patients were selected according to the ICD-10, possibly not all patients with BT of 35 °C or less could be incorporated into this study. Second, trauma patients were also included in this study. We considered that in many cases, elderly people became accidental hypothermia because they could not move after they got injury due to fall, however the detail of trauma was unknown. Third, we obtained SOFA score by using the worst value for each physiological variable within the past 24 h after admission to ICU. Therefore, the time of scoring SOFA was not strictly uniform. Forth, although the target case was a patient with AH requiring ICU management, there was an absence of a protocol for care which was used consistently and contents of the treatment were left to the individual physicians. Furthermore, regarding the details of the cause of death, although there was a record, most of these were inadequate or missing data. Hence, setting a unified treatment indication and contents for patients with AH and considering further research are necessary.

## Conclusions

This study indicated that the SOFA score may be validated for predicting in-hospital mortality among patients with AH admitted to ICU. Therefore, we believe that SOFA score may be useful for risk stratification in order to select appropriate patients for ICU admission.

## Data Availability

No data is available.

## Abbreviations

AH: Accidental hypothermia
AUROC: Area under the receiver operating characteristic curve
BT: Body temperature
CCMC: Critical care medical center
CI: Confidence interval
ECMO: Extracorporeal membrane oxygenation
GCS: Glasgow Coma Scale
ICD-10: International Classification of Diseases, Tenth Revision
ICU: Intensive care unit
IQR: Interquartile range
JCS: Japan Coma Scale
qSOFA: quick SOFA
SIRS: Systemic inflammatory response syndrome
SOFA: Sequential Organ Failure Assessment
